# The Resilience of Microbial Community under Drying and Rewetting Cycles of Three Forest Soils

**DOI:** 10.3389/fmicb.2016.01101

**Published:** 2016-07-19

**Authors:** Xue Zhou, Dario Fornara, Makoto Ikenaga, Isao Akagi, Ruifu Zhang, Zhongjun Jia

**Affiliations:** ^1^State Key Laboratory of Soil and Sustainable Agriculture, Institute of Soil Science, Chinese Academy of SciencesNanjing, China; ^2^University of Chinese Academy of SciencesBeijing, China; ^3^Agri-Food and Biosciences InstituteBelfast, Ireland; ^4^Research Field in Agriculture, Agriculture Fisheries and Veterinary Medicine Area, Kagoshima UniversityKagoshima, Japan; ^5^National Engineering Research Center for Organic-based Fertilizers, Jiangsu Collaborative Innovation Center for Solid Organic Waste Resource Utilization, Nanjing Agriculture UniversityNanjing, China

**Keywords:** rRNA, rDNA, amoA, pyrosequencing, qPCR, forest, seed bank

## Abstract

Forest soil ecosystems are associated with large pools and fluxes of carbon (C) and nitrogen (N), which could be strongly affected by variation in rainfall events under current climate change. Understanding how dry and wet cycle events might influence the metabolic state of indigenous soil microbes is crucial for predicting forest soil responses to environmental change. We used 454 pyrosequencing and quantitative PCR to address how present (DNA-based) and potentially active (RNA-based) soil bacterial communities might response to the changes in water availability across three different forest types located in two continents (Africa and Asia) under controlled drying and rewetting cycles. Sequencing of rRNA gene and transcript indicated that Proteobacteria, Actinobacteria, and Acidobacteria were the most responsive phyla to changes in water availability. We defined the ratio of rRNA transcript to rRNA gene abundance as a key indicator of potential microbial activity and we found that this ratio was increased following soil dry-down process whereas it decreased after soil rewetting. Following rewetting Crenarchaeota-like 16S rRNA gene transcript increased in some forest soils and this was linked to increases in soil nitrate levels suggesting greater nitrification rates under higher soil water availability. Changes in the relative abundance of (1) different microbial phyla and classes, and (2) 16S and *amoA* genes were found to be site- and taxa-specific and might have been driven by different life-strategies. Overall, we found that, after rewetting, the structure of the present and potentially active bacterial community structure as well as the abundance of bacterial (16S), archaeal (16S) and ammonia oxidizers (*amoA*), all returned to pre-dry-down levels. This suggests that microbial taxa have the ability to recover from desiccation, a critical response, which will contribute to maintaining microbial biodiversity in harsh ecosystems under environmental perturbations, such as significant changes in water availability.

## Introduction

Climate change effects on seasonal precipitation regimes and on rates of soil evapotranspiration are predicted to strongly influence soil water availability across forest ecosystems worldwide through its effects on both seasonal precipitation regimes and soil evapotranspiration (Dale et al., 2001; Westerling et al., 2006; Malhi et al., 2008). Seasonal changes in rainfall events might lead to cooler-wetter winters and hotter-drier summers with important consequences for soil nutrient mobilization and soil microbiological processes. In particular large increases in water availability during intense rainfall events occurring at the end of prolonged dry seasons might greatly enhance soil CO_2_ efflux pulses. Such increase in soil respiration is primarily due to enhanced microbial mineralization of carbon (C) and nitrogen (N) organic substrates and is referred to as “Birch Effect” (Birch, 1958; Borken and Matzner, 2009; Inglima et al., 2009). This effect well explains large CO_2_ pulses, which result from rewetting events after dry summer periods and accounts for a significant proportion of annual C budgets in forest ecosystems (Xu and Qi, 2001). Despite soil microbial communities may play a crucial role in mediating the “Birch Effect” it is not clear how soil microbial abundance and community structure will change under drying and rewetting cycles in forest ecosystems.

Previous research studies have mainly focused on the chemical process of microbial mineralization, which is associated with high rates of C (Fierer and Schimel, 2002; Miller et al., 2005) and N release during the dry-wet transition in forest soil ecosystems (Franzluebbers, 1999; Fierer and Schimel, 2002; Mikha et al., 2005). Similar studies show that response patterns of soil microbial biomass to rainfall events could be site-dependent whereby microbial biomass can either increase (Basu et al., 1991; Jia and Insam, 1991; Luizao et al., 1998) or decrease during the rainy season (Singh et al., 1989; Barbhuiya et al., 2004). Microorganisms have an important role in the regulation of ecosystem processes such as nutrient cycling. The abundance and diversity of microbial communities has important implications for the stability and function of nature and semi-natural ecosystems (Hashsham et al., 2000; Bell et al., 2005). So far, field investigations addressing potential responses of soil microorganisms to dry-wet cycles have been limited by the use of techniques with relatively low taxonomic resolution. Recent findings show how the use of clone libraries can help better understanding the response of soil fungal and bacterial communities to climate change (Castro et al., 2010). By using 16S-based denaturing gradient gel electrophoresis (DGGE), Griffiths et al. (2003) have also demonstrated how bacterial communities in grassland soils can be highly resistant to water stress.

Desiccation will contribute to increasing nutrient-poor soil environments with negative effects on the metabolic state of microorganisms as it was shown in both natural communities and laboratory strains (Braeken et al., 2006). Survival of bacterial under desiccation conditions has been studied in different soil environments such as in soils under rhizoremediation (Vilchez and Manzanera, 2011) or in grassland soils (Barnard et al., 2013). In these studies, a large variation of survival times has been observed where for example, Gram-positive bacteria tend to better survive to desiccation than Gram-negative organisms. Changes in specific life strategies of microorganisms were also documented as key response to drought events. However, most of the experimental work so far has been culture-dependent often failing to address the effects of repeated cycles in water availability on microbial dormancy mechanisms in response to desiccation.

Despite changes in microbial community composition and structure under drying and rewetting cycles can greatly influence soil nutrient availability and productivity in forest ecosystems, the net response of soil microorganisms to multiple dry/wet cycles is still poorly understood. Here we suggest that high-taxonomic-resolution techniques can provide detailed phylogenetic-level data (Shi et al., 2015), which could help better understanding the genetic basis of dormancy regulation in soil ecosystems. The proportional change of certain phenotypes could be linked with changes in key variables of the complex soil matrix without the need to use specific biomarkers. High-pyrosequencing based on bacterial microbial DNA and RNA allows the phylogenetic characterization of bacterial groups (rRNA genes), which have the activity of synthetic proteins (rRNA). It has been then suggested that rRNA is a reliable indicator of metabolic state in microbial assemblages (Schippers et al., 2005; Jones and Lennon, 2010; Blazewicz et al., 2013). Thus the main aim of this study is to investigate the potential response of the soil microbial community to extreme desiccation events and subsequent rewetting across three different forest sites. First we used 454 pyrosequencing to analyze ribosomal RNA genes and transcripts from soil samples, which have been exposed to drying-rewetting cycles under different climatic conditions. Second we measured the abundance of selected phylogenetic marker genes (i.e., bacterial and archaeal 16S rRNA and *amoA* gene) and transcripts by performing quantitative PCR. By addressing how soil microbial communities might respond to drying-rewetting cycles, in forest ecosystems, our study contributes to improving our mechanistic understanding of how microorganisms can ultimately deal with significant fluctuations in soil water availability.

## Materials and Methods

### Site Description and Soil Sampling

In our study, we choose three typical forest soils with long distance and similar precipitation pattern, including: (i) African forest soil was collected from Egerton University (0°22′S, 35°55′E), Kenya. The annual mean temperature is 17.7°C, and the annual mean precipitation is 1049 mm. (ii) Chinese forest soil was sampled from the Tea Research Institute of the Chinese Academy of Agricultural Sciences (120°09′E, 30°14′N), Hangzhou City. The annual mean temperature is 17.0°C, and the annual mean precipitation is 1533 mm. (iii) Japanese forest soil was harvested from Minami–Kyushu city, Kagoshima (31°21′E, 130°26′N), Japan. The annual mean temperature is 18.8°C, and the annual mean precipitation is 2400 mm.

At each forest site, three large independent plots were randomly selected under same topography and vegetation type, and three replicate grids were then randomly established within each plot. Then within each grid three replicate soil samples were collected from the A horizon between 0 and 20 cm depth. One composite soil sample was calculated by homogenization of replicated samples after removal of plant residues, roots, stones, and obvious macrofauna. Immediately after transportation to the laboratory, soil samples were sieved through a 2 mm mesh before storage at 4°C until further use. Sub-samples were air-dried for physical and chemical analysis.

### Laboratory Dry-down and Wet-up

**FIGURE 1:**
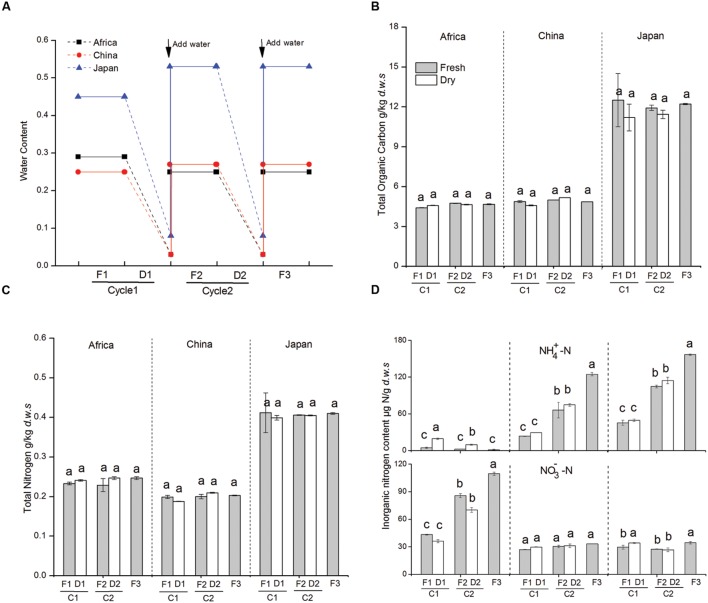
**(A)** Dynamics of soil water content during dry-wet cycles in three experimental soils (D: no water input, F: weekly water inputs). **(B)** Dynamics of soil total soil organic carbon during dry-wet cycles. **(C)** Dynamics of total soil nitrogen during dry-wet cycles. **(D)** Changes in nitrate and ammonium concentration in soil microcosms in response to changes in water availability. Bars indicate standard deviations of triplicate soil samples. Abbreviations represent different treatments: fresh soil (F1), 1st dry-down (D1), 1st wet-up (F2), 2nd dry-down (D2), and 2nd wet-up (F3). The designation C1 represent the first cycle from fresh to dry condition and C2 represent the second cycle from fresh to dry condition.

Soils were exposed to drying and rewetting treatments during the incubation at 28°C. There were 12 replicates for each soil so that soils could be destructively sampled in triplicate after each dry-down and wet-up incubation. The treatment consisted of two dry/wet cycles (**Figure 1A**). One dry/wet cycle started with a 7-days dry-down period, and followed by a 7-days rewetting, which consisted of rewetting to ca. 40% soil maximum water holding capacity (WHC). Soils were incubated in sealed polythene bottles. The soils were dried to constant weight by removing the jar lid and blowing dry air across the soil for 7 days. Soils were rewetted by slowly adding sterile deionized water into the jar using a syringe until the soils reached to 40% WHC, and incubated for 7 days. The total incubation time for the 2-cycle treatment was 28 days.

### Soil Properties

Soil properties were determined as previously described (Zhou et al., 2015). Soil water content was measured at 105°C for 8 h. Soil pH was determined in 2.5:1 (w:v) ratios of soil with distilled water using a DMP-2 mV/pH detector (Quark Ltd, Nanjing, China). Total soil C & N (%) were determined simultaneously by the Dumas method using a LECO CN 2000. NH_4_^+^-N and NO_3_^-^-N were extracted with 2 M KCl at a soil/solution ratio of 1:5 by shaking at 200 rpm for 60 min and determined by a Continuous Flow Analyser (San++System, Skalar, Holland).

### Soil Nucleic Acid Extraction

Soil nucleic acids were extracted from 0.5 g soil using the FastDNA spin kit for soil (Qbiogene, Inc., Irvine, CA, USA) according to the manufacturer’s instructions. DNA purification was conducted with 5.5 M guanidine thiocyanate solution by removing humic substance contamination. The quality and concetration of the extracted DNA was measured by gel electrophoresis (0.8% agarose) and Nanodrop ND-1000 UV-Vis spectrophotometer (NanoDrop Technologies Inc, Wilmington, DE, USA), and stored at -20°C.

RNA was extracted using the protocol of Griffiths et al. (2000) with the modification that glass beads was performed twice. The RNA extraction method is detailed as described in (Ding et al., 2015). rRNA was purified by the RNeasy mini Kit (Qiagen, Hilden, Germany) according to the manufacturer’s instructions, and quantified using UV-vis Spectrophotometer (ND-1000 NanoDrop). otal RNA was converted to cDNA using PrimeScript 1st Strand cDNA Synthesis Kit (TaKaRa) using random hexamers, and stored at -20°C for amplicon sequencing.

### Pyrosequencing Analysis of 16S rRNA Gene

Pyrosequencing of total 16S rRNA genes of the V4 regions was carried out on the Roche 454 GS FLX Tianium sequencer (Roche Diagnostics Corporation, Branford, CT, USA) using the putatively universal primer pairs of Tag-515F and Tag-907R (**Supplementary Table S1**), as described previously (Xia et al., 2011). The PCR reaction performed in a thermal cycler (Bio-Rad Laboratories, Hercules, CA, USA) under the following procedure: 94°C for 5 min, followed by 30 cycles of 94°C for 30 s, 55°C for 30 s, and 72°C for 55 s, with a 10 min extension at 72°C (**Supplementary Table S1**). The reaction mixture consisted of 0.2 mM of each deoxynucleoside triphosphate, 1 × PCR buffer (Mg^2+^ Plus), 0.1 μM of each primer, 1.25 U of TaKaRa Taq HS polymerase (TaKaRa Biotech, Dalian, China), and 50 ng of soil DNA template in a final volume of 50 μl. PCR products were checked by 1.2% agarose gels, and purified using the Agarose Gel DNA Purification Kit (TaKaRa), and then the PCR amplicons were combined in equimolar ratios into a single tube in preparation for pyrosequencing analysis with soil samples from each treatment.

The pyrosequencing data of 16S rRNA genes were analyzed using the Quantitative Insights Into Microbial Ecology (QIIME) soft package (Caporaso et al., 2010), and sequences below quality score of 25 or 200 bp in length were excluded before further analysis. A total of 310, 308 high-quality sequence reads of 16S rRNA gene were obtained with an average length of ∼390 bp (**Supplementary Table S2**). All samples were rarefied to 2,300 sequences for statistical analysis including rarefaction curves of Shannon diversity (**Supplementary Figure S1**). The taxonomic identity of each phylotype was determined using ribosomal database project (RDP) Classifier with a confidence threshold of 0.80 (Cole et al., 2005). Finally, sequences were clustered into operational taxonomic units (OTUs) based on 97% sequence similarity with UCLUST (Edgar, 2010). Based on the sequences and/or OTUs obtained, microbial diversity, and richness analyses, principal component analysis, and canonical correspondence analysis were performed. The Crenarchaeota-like 16S rRNA genes were screened by RDP classifier at phyla level (Cole et al., 2009), while bacterial ammonia oxidizers of *Nitrosococcus* and *Nitrosospira* and nitrite oxidizers of *Nitrobacter* and *Nitrospira* were assigned at the genus level for further analysis.

### Population Size

The abundance of genes and transcripts were assessed by quantitative polymerase chain reaction (qPCR) on a CFX96 Optical Real-Time Detection System (Bio-Rad Laboratories, Inc. Hercules, CA, USA). The population size of bacterial and archaeal communities was assessed by quantification of 16S rRNA gene copies in soil samples using the primer pairs 515F–907R (Stubner, 2002) and A364aF–A934bR (Kemnitz et al., 2005), respectively. AOA and AOB in soil samples were quantified with the primer pairs of Arch-amoAF/Arch-amoAR (Francis et al., 2005) and amoA-1F/amoA-2R (Rotthauwe et al., 1997), respectively.

The DNA and cDNA templates were 10-fold diluted to 1–10 ng in each reaction mixture. Quantitative PCR reactions were carried out in 20-μL reaction mixtures containing 10 μL 2x SYBR Premix Ex Taq (Takara Biotech, Dalian, China), 200 nM of each primer, and 20 ng DNA template. The thermal program for the real-time PCR assay was shown in **Supplementary Table S1**. Standard curves, spanning 10^7^–10^1^, were constructed by dilution series of plasmids harboring the aimed gene. The amplification efficiency ranges from 90 to 115% and *R*^2^ values were approximately 0.997–0.999 in each reaction. Special amplification of target genes was confirmed by melting curve analysis always resulting in a single peak.

### Statistical Analysis

Statistical analyses of pyrosequencing were performed in R software (Version 3.0.2, vegan package). We performed analysis of variance (ANOVA) to test for potential significant effects of soil moisture on the abundance of 16S rRNA genes and transcripts across our microbial communities. We performed multiple Student–Newman–Keuls tests to identify significant differences between each treatment. We also used MRPP, ANOSIM, and ADONIS to test for group difference by using R software. All data analyses were performed using SPSS version 10.0 (IBM Co., Armonk, NY, USA). Differences at *P* < 0.05 were considered statistically significant. The pyrosequencing reads of the total 16S rRNA genes have been deposited in the European Nucleotide Archive under the accession number Hx2000053322.

## Results

### Soil C and N Pools under Drying-rewetting Cycles

We found that soil organic C and N content did not change during the dry-down and wet-up treatments across the three forest sites (**Figures 1B,C**). Soil NH_4_^+^ and soil NO_3_^-^ pools significantly responded to changes in water availability (**Figure 1D**). In particular, NH_4_^+^ levels significantly increased with greater water availability in soils collected from Asian (Chinese and Japanese) forest sites (from 23.48 to 124.65 ppm and from 45.28 to 156.77 ppm, respectively). However, soil NO_3_^-^ levels were not significantly affected by changes in water availability in Chinese soil and soil NO_3_^-^ was slightly increased in the second wet-up in Japanese soil. Soil NH_4_^+^ and NO_3_^-^ pools followed the opposite pattern in the African (AS) forest soils. Here soil NH_4_^+^ increased on average by 336% with repetitive dry-down treatments, and declined by 87.5% after repetitive wet-up treatments, whereas large amounts of NO_3_ accumulated during repetitive wet-up (+97.1%) but decreased after dry-down treatment (by average -17.4%).

### Quantification of Bacterial and Archaeal 16S rRNA Genes and Transcripts

**FIGURE 2:**
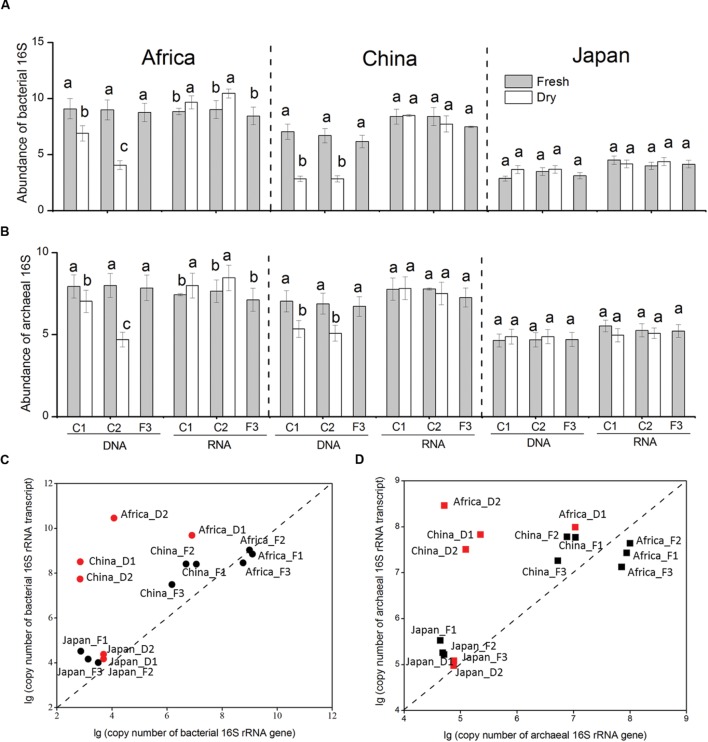
**Changes in the abundance of bacterial **(A)** and archaeal **(B)** 16S rRNA genes and transcripts in response to dry-down and wet-up treatments on the three forest soil types.** Correlation of abundance of bacterial **(C)** and archaeal **(D)** 16S rRNA gene and transcript. The abundances were log-transformed to reduce skewness of the data. Red points represent dry-down treatment, and black points represent pre-dry-down and rewetting treatment. The designation AF1 represents the fresh African forest soil. The Africa D1 represents the 1st dry-down treatment of African forest soil. The designation Africa_F2 represents the 2nd wet-up treatment of African forest soil. The original data is shown in **Supplementary Table S6**.

Population size of bacterial and archaeal 16S genes (DNA) and transcripts (RNA) differed among sites over dry-down and wet-up treatments (**Figures 2A,B**). In the African and Chinese forest soils repetitive dry-down treatments decreased the overall abundance of bacterial and archaeal 16S rRNA gene. In contrast, repetitive wet-up stimulated the abundance of bacterial and archaeal 16S rRNA gene back to the original level. However, neither the abundance of bacterial nor archaeal 16S rRNA genes was significantly affected by the change of water content in the Japanese soil.

In our study, we define rRNA transcipt: rRNA gene ratio as the indicator of potential activity of microorganisms (**Figures 2C,D**), as previous investigations (Kemp et al., 1993; Kerkhof and Ward, 1993; Poulsen et al., 1993; Muttray et al., 2001). In the African and Chinese soil, the bacterial rRNA transcript: bacterial rRNA gene ratio and archaeal rRNA transcript: archaeal rRNA gene ratio both stimulated by dry-down and declined by wet-up. However, neither the bacterial ratio nor the archaeal ratio was influenced by the change of water availability in Japanese soil.

### Quantification of Ammonia-oxidizing Microbes

**FIGURE 3:**
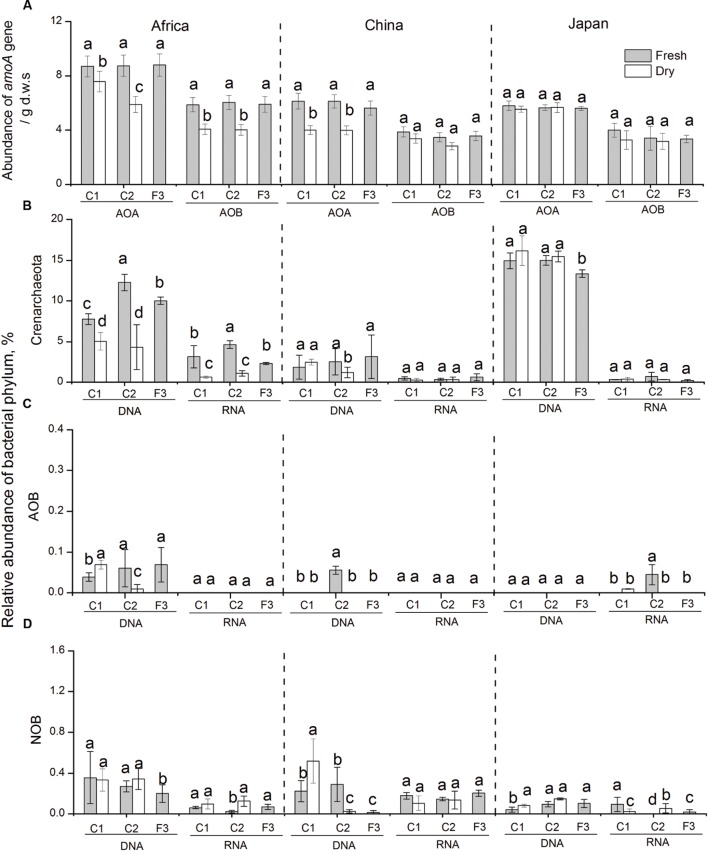
**(A)** Changes in the abundance of bacterial and archaeal *amoA* genes in response to dry-down and wet-up treatments on the three forest soil types. Changes in the relative abundance of Crenarchaeota (AOA) **(B)**, AOB **(C)**, and NOB **(D)** in soil microcosms in response to changes in water availability. Bars indicate standard deviations of triplicate soil samples. All the designations are as the same as above.

Real-time quantitative PCR was also employed to address changes in population size of AOA and AOB by quantifying archaeal and bacterial *amoA* genes (**Figure 3A**). The abundance of archaeal and bacterial *amoA* genes showed different response patterns to changes in water availability across the three forest sites. In African and Chinese soils, archaeal *amoA* genes significantly decreased with repetitive dry-down treatments (average -96.2 and -99%, respectively), and increased to the original level with the repetitive wet-up treatments. However, neither dry-down nor wet-up affected the abundance of AOA in Japanese soil (*P* > 0.05). Bacterial *amoA* gene was generally 2 to 3 orders magnitude lower than archaeal *amoA* genes across different soils. Bacterial *amoA* gene decreased with repetitive dry-down treatments (average -99%), and significantly increased to original levels under repetitive wet-up treatments (*P* < 0.05) in AS. In contrast, no detectable dry-down-related or wet-up-related changes in bacterial *amoA* gene were found in Chinese and Japanese soils, suggesting a marked resistance to changes in water availability.

### Response of Microbial Community Structure

A total of 309,840 high-quality sequence reads of DNA and cDNA were obtained with length of ∼390 bp (**Supplementary Table S1**) with an average ± SE of 3443 ± 473 in each sample. Rarefaction curves of Shannon diversity index were used to estimate microbial diversity among dry-rewetting cycles in each plot (**Supplementary Figure S1**; **Supplementary Table S3**). All rarefaction curves reached a plateau indicating that sequence-derived diversity in this study was sufficient to characterize microbial species in each soil. Present (DNA-based) and potentially active (RNA-based) microbial diversities at each site responded differently to changes in water availability. In African and Japanese soil, present (DNA-based) microbial diversity remained stable under both dry-down and wet-up treatments. However, potentially (RNA-based) active microbial diversity decreased under both dry-down and rewetting treatments in these two soils. On the contrary, present (DNA-based) microbial diversity increased significantly as dry-down progressed in Chinese soil, then returned to the original diversity after rewetting. Potentially (RNA-based) active diversity displayed remarkable resistance to drying and rewetting cycles.

**FIGURE 4:**
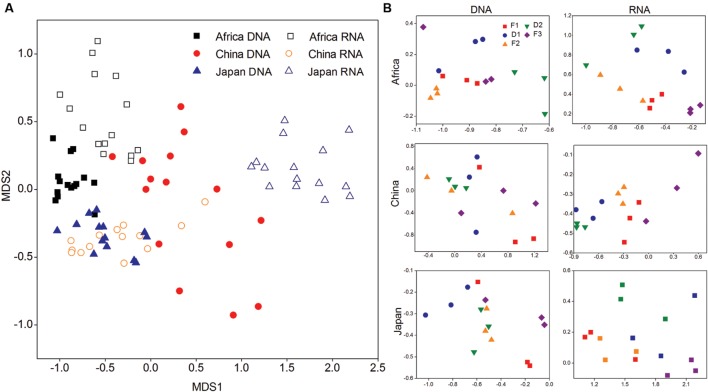
**(A)** The compositional structure of 16S rRNA genes and transcripts among three forest soils described by non-metric multi-dimensional scaling (NMDS) plots. **(B)** The change pattern of bacterial 16S rRNA genes and transcripts in response to dry-down and wet-up treatments described by NMDS plots.

**Figure 4** shows shifts in the microbial communities during the change of water availability in non-metric multidimensional scaling (NMDS) plots of the pairwise UniFrac distance ordinations. We found that F1, F2, F3 were clustered and D1, D2 were clustered in DNA of Chinese soil, and in RNA of African and Japanese soil (*P* < 0.05) (**Supplementary Table S4**).

### Response of Bacterial Groups and Nitrifying Communities to Dry-down and Wet-up

#### Bacterial 16S rRNA Gene

The relative abundance of bacteria groups was analyzed by aggregating all taxonomic classification at the phylum and class levels. Soil bacterial communities at the three forest sites were dominated in relative abundance by the Actinobacteria phylum (mostly Actinobacteria class), and the Proteobacteria phylum (primarily alpha, beta, gamma, and delta class). Although the bacterial community structure was not significantly influenced by dry-down and wet-up, the relative abundance of the different bacterial phyla and class followed different response patterns to changes in water availability (**Table 1**, **Supplementary Table S5**).

**Table 1:** Significant changes (%) in bacterial phylotypes at the taxonomic level of classes (*P* < 0.05) affected by the dry-down and wet-up.

		Phylum	Class	F1 → D1	D1 → F2	F2 → D2	D2 → F3
Africa	DNA	Actinobacteria	Actinobacteria	4.9	-2.6	2.5	-0.3
		Proteobacteria	Alphaproteobacteria	1.1	-1.3	1.9	-1.5
		Firmicutes	Bacilli	1.0	-1.1	0.8	-0.4
		Verrucomicrobia	Opitutae	0.0	-0.2	0.0	0.0
	RNA	Actinobacteria	Actinobacteria	11.1	-4.1	-1.3	-8.2
		Chloroflexi	Ktedonobacteria	-0.6	3.1	-0.3	0.7
		Acidobacteria	Acidobacteria_Gp1	-1.4	0.1	-2.2	2.5
		Planctomycetes	Planctomycetacia	-4.3	6.5	-7.3	8.3
China	DNA	Proteobacteria	Alphaproteobacteria	2.4	-3.7	4.5	-1.9
		Proteobacteria	Deltaproteobacteria	0.7	-1.0	1.1	-1.8
		Proteobacteria	Betaproteobacteria	-2.9	3.0	-0.2	7.8
		Proteobacteria	Gammaproteobacteria	-7.1	6.6	-9.4	16.0
		Planctomycetes	Planctomycetacia	5.0	-2.0	5.5	-2.5
		Verrucomicrobia	Subdivision3	0.6	-0.6	0.1	-0.1
		Acidobacteria	Acidobacteria_Gp1	2.4	-1.6	0.6	-1.9
		Acidobacteria	Acidobacteria_Gp3	1.3	-0.8	0.2	-0.6
		Acidobacteria	Acidobacteria_Gp6	0.6	-1.5	0.1	-1.0
		Acidobacteria	Acidobacteria_Gp7	0.4	-0.1	0.2	-0.7
	RNA	Actinobacteria	Actinobacteria	9.7	-2.3	1.8	-8.7
		Planctomycetes	Planctomycetacia	3.0	-0.7	1.9	-6.2
		Acidobacteria	Acidobacteria_Gp3	-0.6	0.5	-0.6	0.4
		Acidobacteria	Acidobacteria_Gp6	-0.6	0.1	-0.4	0.9
		Acidobacteria	Acidobacteria_Gp1	-0.1	0.1	-0.1	0.1
		Acidobacteria	Acidobacteria_Gp4	0.0	0.8	-1.0	1.6
		Proteobacteria	Deltaproteobacteria	-2.0	0.7	-1.0	3.3
		Proteobacteria	Gammaproteobacteria	-2.1	0.1	-0.6	2.1
		Proteobacteria	Betaproteobacteria	-2.3	0.5	-1.6	2.1
		Proteobacteria	Alphaproteobacteria	-6.4	4.0	-4.6	9.4
Japan	DNA	Actinobacteria	Actinobacteria	4.4	-3.3	2.0	-10.2
		Proteobacteria	Betaproteobacteria	-1.1	0.8	-0.7	7.6
		Proteobacteria	Gammaproteobacteria	-1.0	0.7	-1.4	5.0
	RNA	Proteobacteria	Deltaproteobacteria	0.1	-0.2	5.3	-5.7
		Proteobacteria	Gammaproteobacteria	-2.6	14.7	-8.2	11.9
		Proteobacteria	Betaproteobacteria	-0.3	5.8	-0.5	9.7
		Firmicutes	Bacilli	4.3	-3.6	10.8	-11.3
		Planctomycetes	Planctomycetacia	0.2	-0.1	0.9	-0.6
		Chloroflexi	Anaerolineae	-2.0	1.1	-0.8	0.1
		Acidobacteria	Acidobacteria_Gp4	-1.2	0.3	-1.3	0.3
		Acidobacteria	Acidobacteria_Gp1	-0.9	0.0	-0.4	0.2


*The designation of F1 → D1 means the relative abundance of F1 treatment minus the relative abundance of D1 treatment.*

Overall, there are 5, 4, and 2 phyla were significantly influenced by repeated dry-down and wet-up treatments in the African soil, Chinese soil and Japanese soil, respectively. Actinobacteria were the most responsive phylum to changes in water availability at African and Japanese sites. Actinobacteria increased with dry-down and decreased with wet-up. The relative abundance of Verrucomicrobia in African and Chinese soils followed a pattern similar to Actinobacteria. Additionally, Firmicutes (Africa), Acidobacteria (China), and Planctomycetes (China) phyla also increased with dry-down and decreased with wet-up treatments. The other main responsive phylum was Proteobacteria, which responded in different ways. Our data shows that dry-down decreased the relative abundance of Proteobacteria (mainly Betaproteobacteria and Gammaproteobacteria class) in Chinese and Japanese soils whereas wet-up increased their abundance. Proteobacteria (mainly Alphaproteobacteria class) in African soil showed the opposite response pattern to the dry-down and wet-up treatments. At the class level, Betaproteobacteria and Gammaproteobacteria were highly responsive to changes in water availability and decreased under dry-down and wet-up treatments at Chinese and Japanese sites.

#### Bacterial 16S rRNA Transcript

In our study, changes in the relative abundance of rRNA appeared to reflect changes in potential microbial activity. Each soil has 4 phyla which were significantly influenced by the change of water availability. The community-dominating Actinobacteria transcripts were similar in African and Chinese soils and increased with dry-down and decreased with wet-up treatments. Similarly, the Firmicutes phylum followed the Actinobacteria pattern in Japanese soil. In contrast, Acidobacteria showed the opposite change pattern in Chinese and Japanese soils, which declined with dry-down and accumulated with wet-up. Likewise, Chloroflexi transcripts followed patterns similar to that of Acidobacteria. At the class level, Alphaproteobacteria (China) and Gammaproteobacteria and Betaproteobacteria (Japan) were the most responsive class in Chinese and Japanese soils, respectively, and decreased with dry-down and increased with wet-up cycles. Interestingly, the Bacilli class significantly increased under dry-down and sharply decreased with wet-up treatment in Japanese soils.

#### Nitrifying Communities

Proportional changes in nitrifying population were assessed by screening 16S rRNA gene and transcript sequences of some dominant phylotypes in ammonia oxidizers and nitrite oxidizers (**Figures 3B–D**). Among crearchaeota, we observed a decline in the proportion of crearchaeota with dry-down treatments (from 8 to 5%, and 12 to 4%, respectively), and an increase under wet-up treatments (from 5 to 12%, and 4 to 10%, respectively) in AS, which is consistent with the result from quantitative PCR. However, in Chinese and Japanese soils, we were unable to detect a significant change in crearchaeota. Additionally, no detectable change in relative abundance of AOB and NOB were observed in the experimental soils. At the transcript level, the increase in the relative expression of crearchaeota-16S rRNA was generally consistent with trends at the DNA level, that the proportion of crearchaeota decreased with dry-down (from 3 to 1%, and 5 to 1%, respectively), and increased with wet-up (from 1 to 5%, and 1 to 3%, respectively) in the African soils. Similarly, in Chinese and Japanese soils, three nitrifying microbes (crearchaeota, AOB, and NOB), with extremely low transcript abundance, showed no response to changes in water availability.

## Discussion

Overall our findings demonstrate that the microbial community of three different forest soils shows high desiccation-tolerance and high resilience to changes in soil water availability. To estimate the state of activity of our soil microorganisms we calculated the rRNA concentration per cell by computing rRNA:rRNA gene ratios (Jones and Lennon, 2010; DeAngelis et al., 2011). We found significantly higher bacterial and archaeal rRNA: rDNA ratios in response to desiccation in our African and Chinese. This suggests an increased accumulation of ribosome concentration when microorganisms face a drought stress. There are organisms that can survive desiccation by entering inactive metabolic states (Vilchez and Manzanera, 2011 #1854; Jones and Lennon, 2010 #1671; Oliver, 2010 #1851), which are associated with the reduced concentrations of nucleic acids (Choi et al., 1996; Dell’Anno et al., 1998; Lebaron et al., 2001; Suzina et al., 2004). Increased ribosome under water stress can provide microorganisms with a higher protein synthesis potential when environmental conditions improve again (Sukenik et al., 2012). In additionally, we found that the change in water availability did not influence the bacterial and archaeal rRNA: rDNA ratios at the forest sites in Japan. Such variability in microbial response to water availability among our study sites could be explained by the effects that different local factors may have on microbial life-strategies under environmental perturbations. For example, the organic carbon and nitrogen content was significantly higher in the Japanese soils and may provide large reservoirs of organic substrate, which can support energy requirements for microorganisms, and prevent resource starvation when facing the desiccation stress. Additionally, a significant proportion of organic carbon might be preferentially mineralized upon rewetting, and thus limit decreases in bacterial and archaeal 16S rRNA genes. Finally, it is possible that the different response patterns of microorganisms to water availability partly depend on changes in total microbial community structure and composition as shown in **Figure 4**.

The response patterns of bacterial phyla and classes are both site-specific and taxa-specific across our experimental forest soils. For example, Proteobacteria (the most abundant phylum of the present and potentially active community), displayed a water-related opportunistic pattern at the DNA level in soils collected from Chinese and Japanese forest sites. In particular, the relative abundance of Proteobacteria declined with desiccation while increased after rewetting. In contrast, Proteobacteria increased in relative abundance with desiccation, and decreased with rewetting at the DNA level in soils collected from African forest sites. Such variability could be explained by differences in response patterns at the class level whereby Betaproteobacteria were dominant at the Chinese and Japanese sites whereas Alphaproteobacteria were dominant in the African soils. Previous studies show how the abundance of Betaproteobacteria is positively correlated with C mineralization rates (Fierer et al., 2007), which increases with rewetting of dry soils (Birch, 1958; Borken and Matzner, 2009; Inglima et al., 2009). Another important phylum, Actinobacteria, was either stimulated by desiccation or reduced by rewetting at the DNA level in the African and Japanese soils. This agrees with the response of bacterial communities in a California grassland soil study (Barnard et al., 2013). Interestingly, Actinobacteria followed the same pattern at the RNA level in African and Chinese soils. Increases in Actinobacteria during dry-down periods may be attributed to the preparation for next nutrient acquisition. Previous studies have demonstrated that large members of the Gram-positive, high G+C content Actinobacteria phylum were drought resistant and able to grow under extrem dry conditions (Goodfellow and Williams, 1983; Chowdhury et al., 2009). Acidobacteria abundance at the RNA level across Chinese and Japanese soils showed an opposite pattern compared to Actinobacteria. This would agree with clear life-strategy differences between these two groups where Actinobacteria are considered copiotrophic (analog to r-strategist: fast growing and highly variable population size) and Acidobacteria are oligotrophic (analog to k-strategist: slow growing and stable population size). Additionally, beyond potential differences in life-resource strategies our findings clearly suggest that the response of key bacterial phyla and classes to changes in water availability was driven by niche differentiation. Microbial responses to environmental stress may be taxa-specific and dependent on changes in space dimension. Because relationships between rRNA concentration and growth rate can differ significantly among taxa (Barnard et al., 2013), relative rRNA abundance of each phylum may not provide convincing information about which taxa are more active following changes in water availability.

We found that nitrification processes were negligible under dry-down treatments, mainly because nitrifies remain “inactive” under dry soil conditions (Placella and Firestone, 2013). Following water additions, however, we observed high rates of gross N mineralization, which is consistent with previous findings (Birch, 1964; Hungate et al., 1997; Saetre and Stark, 2005). These N mineralization responses were different among all the three forest sites. African forest soils showed large NO_3_^-^ pulses following rewetting as was found in previous studies (Holloway et al., 1998; Baron et al., 2009), and this increased nitrification was coupled with significant increases in crearchaeota 16S transcripts. On the contrary, little NO_3_ accumulation was observed in Chinese and Japanese forest soils. No detectable changes were observed either of the abundance of crearchaeota, AOB and NOB 16S transcripts. Such differences in response patterns of nitrifiers among forest soils, might be due to variation in soil gross nitrification rates and soil heterogeneity. The activity of AOA in the nitrification process of African soils confirms findings from the previous studies that AOA might prefer the ammonia released from mineralization (Gubry-Rangin et al., 2010; Zhang et al., 2010). The lower relative abundance of AOA transcripts in Chinese and Japanese soils might relate to negligible nitrification activity during the changes in water availability. The difference in the relative abundance of AOA transcripts might be attributed to variation in soil pH values, with African soils having soil pH (pH 6.0) significantly higher than the others (Chinese soil: pH 4.1; Japanese soil: pH 4.5). Finally, gene and transcript abundances of bacterial and archaeal 16S rRNA and the *amoA* gene abundance of AOA and AOB also returned to pre-dry-down abundance after water additions.

Despite the different response patterns of microbial taxa to the changes in water availability, our evidence is that upon rewetting, the present and potentially active bacterial community structure as well as the abundance of bacterial (16S), archaeal (16S), and ammonia oxidizers (amoA), all returned to pre-dry-down levels. This resilience ability may greatly contributes to the maintenance of microbial diversity in the forest ecosystem soils under future environmental change.

## Author Contributions

XZ and ZJ contributed to the experimental design of the study. XZ collected conducted the analysis of results and wrote the manuscript. All authors contributed to writing and reviewing the manuscript.

## Conflict of Interest Statement

The authors declare that the research was conducted in the absence of any commercial or financial relationships that could be construed as a potential conflict of interest.
