# Supplementary material for: The Resilience of Microbial Community under Drying and Rewetting Cycles of Three Forest Soils
Source: Front Microbiol. 2016 Jul 19;7:1101. doi: 10.3389/fmicb.2016.01101 (PMC4949271; doi:10.3389/fmicb.2016.01101)
Supplement: Supplementary file 1 [file Presentation_1.PDF]

Supplementary Material for

The resilience of microbial community under drying and rewetting cycles of three forest soils

Xue Zhou[1,2], Dario Fornara[3], Makoto Ikenaga[4], Isao Akagi[4], Ruifu Zhang[5], Zhongjun Jia[1,*]

[1]State Key Laboratory of Soil and Sustainable Agriculture
Institute of Soil Science, Chinese Academy of Sciences
Nanjing, 210008, Jiangsu Province, China

[2]University of Chinese Academy of Sciences
Beijing 100049, China

[3] Agri-Food and Biosciences Institute
Newforge Lane, Belfast BT9 5PX, N. Ireland

[4]Research Field in Agriculture,
Agriculture Fisheries and Veterinary Medicine Area,
Kagoshima University, 1–21–24, Korimoto, Kagoshima, 890–0065, Japan

[5]Nanjing Agriculture University
Nanjing, 210006, Jiangsu Province, China

[*]Correspondence should be addressed to Zhongjun Jia at

E-mail: jia@issas.ac.cn

Tel: +86-25-8688-1311, Fax: +86-25-8688-1000

This file includes:

Supplementary Figure S1

Supplementary Table S1-S6

Supplementary Reference

Supplementary Figure Legends

Figure S1.Rarefaction curves of Shannon diversity index on the basis of 16S rRNA genes over dry-down and wet-up in the experimental sites. Data were produced by pyrosequencing analysis. Data was also shown in Table S3.

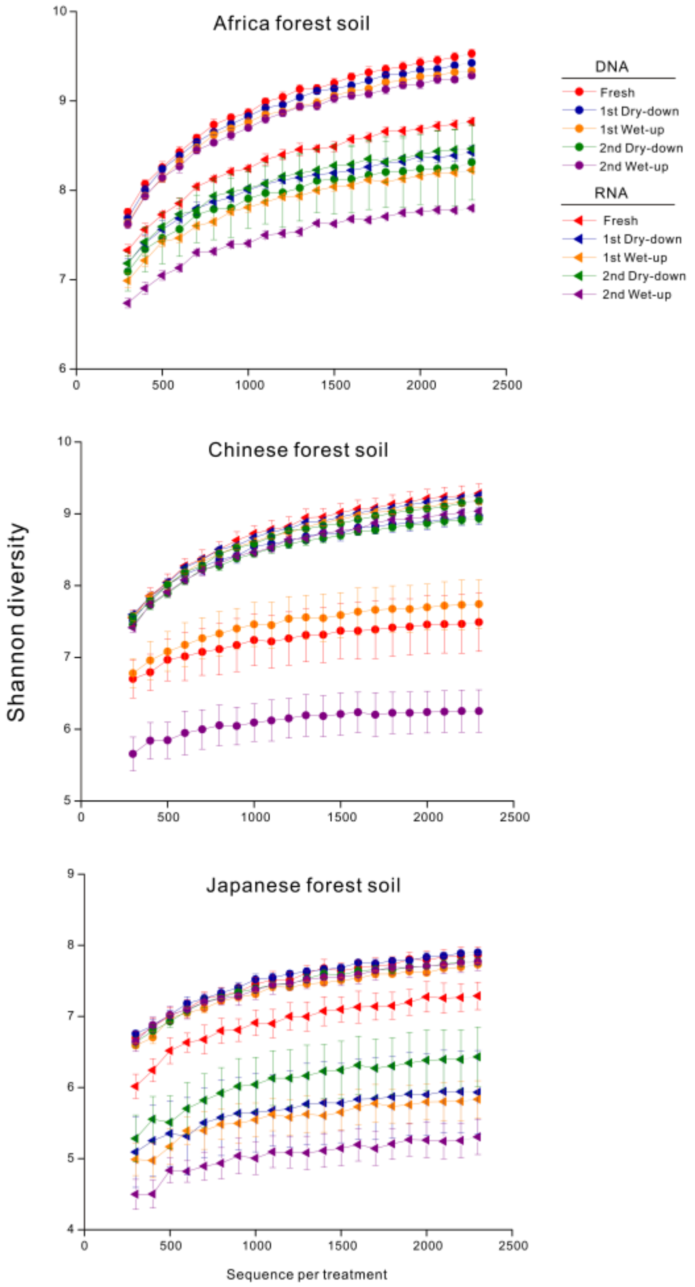

Table S1. Primers and conditions used in this study

Primer Name	Primer sequence (5'-3')	Target gene	Thermal Profile	Molecular analysis	Reference
Arch-*amo*AF	STA ATG GTC TGG CTT AGA CG	archaeal *amoA* gene	95 ℃, 3min; 35 ×(95 ℃, 30s; 55 ℃, 30s; 72 ℃, 30s with plate read); Melt curve 65.0 ℃ to 95.0 ℃, increment 0.5℃, 0:05+ plate read	Real-Time PCR in Fig. 4	(Francis et al. 2005)
Arch-*amo*AR	GCG GCC ATC CAT CTG TAT GT				
amoA-1F	GGG GTT TCT ACT GGT GGT	bacterial *amoA* gene	95 ℃, 3min; 35 ×(95 ℃, 30s; 55 ℃, 30s; 72 ℃, 30s with plate read); Melt curve 65.0 ℃ to 95.0 ℃, increment 0.5℃, 0:05+ plate read	Real-Time PCR in Fig. 4	(Rotthauwe et al. 1997)
amoA-2R	CCC CTC KGS AAA GCC TTC TTC				
515F	GTG CCA GCM GCC GCG G	universal 16S rRNA genes	94 ℃, 5min; 32 ×(94 ℃, 30s; 54 ℃, 30s; 72 ℃, 45s); 72℃,10min; hold at 4℃	454 Pyrosequencing and real-Time PCR in Fig.3	(Stubner 2002)
907R	CCG TCA ATT CMT TTR AGT TT				
A364aF	ACG GTG AGG GAT GAA AGCT	archaeal 16S rRNA gene	95 ℃, 3min; 35 ×(95 ℃, 30s; 55 ℃, 30s; 72 ℃, 30s with plate read); Melt curve 65.0 ℃ to 95.0 ℃, increment 0.5℃, 0:05+ plate read	Real-Time PCR in Fig. 3	(Kemnitz et al. 2005)
A934b	GTG CTC CCC CGC CAA TTC CT				

Table S2. Pyrosequencing summary of the total 16S rRNA genes in the total DNA of the experimental soils tested.

Site	Treatment	R*	DNA		RNA	
			High Quality Read	Bacterial Read	High Quality Read	Bacterial Read
Africa	In situ	1	4133	3757	3990	3824
		2	2907	2699	3790	3618
		3	3533	3266	3822	3782
	1st Dry-down	1	3769	3482	3979	3956
		2	3453	3205	3578	3451
		3	3455	3108	3973	3937
	1st Wet-up	1	3560	3103	3548	3393
		2	3951	3408	3175	3038
		3	3088	2751	3221	3042
	2nd Dry-dow	1	3010	2986	3390	3335
		2	3533	3380	3964	3591
		3	3638	3328	3634	3606
	2nd Wet-up	1	3652	3253	3225	3144
		2	3817	3423	3477	3401
		3	3096	2802	3499	3459
China	In situ	1	2793	2709	3635	3171
		2	2860	2841	3554	2891
		3	2858	2726	3523	3488
	1st Dry-down	1	2993	2921	3223	2625
		2	3092	2995	3031	2555
		3	2966	2877	3673	2985
	1st Wet-up	1	3201	3190	3581	3049
		2	3707	3591	3331	2753
		3	3538	3355	3397	2904
	2nd Dry-dow	1	2744	2710	3697	3021
		2	2493	2430	3730	3015
		3	2541	2519	3202	2599
	2nd Wet-up	1	2389	2193	3675	3296
		2	2544	2544	3997	3809
		3	2419	2367	3674	3139
	Total		95733	89919	107188	97877

* represents replicate, and NA denotes not applicable

Table S2.*(continued)* Pyrosequencing summary of the total 16S rRNA genes in the total DNA of the experimental soils tested.

Site	Treatment	R*	DNA		RNA	
			High Quality Read	Bacterial Read	High Quality Read	Bacterial Read
Japan	In situ	1	3396	2843	3882	3849
		2	3265	2821	3552	3532
		3	3503	2946	3614	3596
	1st Dry-down	1	3988	3187	5628	5538
		2	3800	3237	3258	3168
		3	3233	2673	3580	3518
	1st Wet-up	1	3738	3203	3874	3816
		2	3209	2708	3338	3272
		3	3313	2798	3740	3619
	2nd Dry-dow	1	3199	2690	2715	2694
		2	3815	3193	3985	3966
		3	3956	3378	2377	2347
	2nd Wet-up	1	3625	3502	3304	3246
		2	3488	3149	3502	3468
		3	3470	3148	3572	3553
	Total		52998	45476	53921	53182

* represents replicate, and NA denotes not applicable

Table S3. Shannon diversity index on the basis of 16S rRNA genes over dry-down and wet-up in the experimental sites.

	Treatment	Shannon		
		Africa	China	Japan
DNA	F1	9.53	7.49	7.86
	D1	9.42	8.97	7.90
	F2	9.34	7.74	7.73
	D2	8.31	8.94	7.77
	F3	9.28	6.25	7.77
RNA	F1	8.77	9.28	7.86
	D1	8.43	9.25	7.90
	F2	8.23	9.18	7.73
	D2	8.47	9.18	7.77
	F3	7.80	9.04	7.77

Table S4. MRPP, ANOSIM and ADONIS test group difference by using R software in our study.

		DNA	RNA
		F and D	F and D
Africa	ANOSIM	0.254	0.001*
	ADONIS	0.133	0.005*
	MRPP	0.190	0.030*
China	ANOSIM	0.002*	0.183
	ADONIS	0.001*	0.074
	MRPP	0.001*	0.132
Japan	ANOSIM	0.199	0.023*
	ADONIS	0.144	0.024*
	MRPP	0.119	0.035*

Table S5. Significant changes (%) in bacterial phylotypes at the taxonomic level of phylums (P<0.05) affected by the dry-down and wet-up. The designation of F1→D1 means the relative abundance of F1treatment minus that of D1 treatment.

		Phylum	F1→D1	D1→F2	F2→D2	D2→F3
Africa	DNA	Actinobacteria	4.9	-3.2	0.2	-0.4
		Proteobacteria	2.6	-2.7	3.4	-2.7
		Firmicutes	0.9	-1.1	1.7	-0.5
		Verrucomicrobia	0.5	-0.5	0.3	-0.4
		Crenarchaeota	-4.5	4.2	-8.0	5.7
	RNA	Actinobacteria	11.1	-4.1	1.9	-11.4
		Planctomycetes	-4.5	6.5	-7.3	8.3
		Crenarchaeota	-1.6	3.1	-1.0	1.8
		Chloroflexi	-0.6	3.2	-0.3	0.8
China	DNA	Acidobacteria	11.2	-11.8	2.1	-3.8
		Planctomycetes	5.1	-1.7	5.3	-2.2
		Verrucomicrobia	0.8	-0.8	0.4	-0.1
		Proteobacteria	-4.8	5.3	-4.3	8.4
	RNA	Actinobacteria	9.7	-2.3	1.8	-8.7
		Planctomycetes	2.9	-0.2	1.8	-5.4
		Acidobacteria	-1.4	1.3	-2.1	3.1
		Proteobacteria	-8.4	5.4	-8.1	17.5
Japan	DNA	Actinobacteria	4.4	-3.3	2.0	-10.2
		Proteobacteria	-8.3	6.2	-0.3	11.6
	RNA	Firmicutes	4.6	-3.3	10.3	-11.4
		Planctomycetes	0.1	-0.1	0.8	-0.6
		Chloroflexi	-2.1	1.3	-1.1	0.3
		Acidobacteria	-6.3	1.3	-1.9	1.9

Table S6. Changes in the abundance of bacterial and archaeal 16S and *amoA* genes in response to dry-down and wet-up treatments on the three forest soils.

Target gene	site	DNA					RNA				
		F1	D1	F2	D2	F3	F1	D1	F2	D2	F3
Bacteria	Africa	1.26E+09	8.02E+06	9.89E+08	1.18E+04	5.76E+08	7.19E+08	4.86E+09	1.07E+09	2.88E+10	2.85E+08
	China	1.16E+07	7.04E+02	4.92E+06	6.91E+02	1.50E+06	2.52E+08	3.21E+08	2.56E+08	5.49E+07	3.12E+07
	Japan	7.39E+02	4.89E+03	3.13E+03	4.95E+03	1.35E+03	3.30E+04	1.49E+04	1.01E+04	2.37E+04	1.45E+04
Archaea	Africa	8.70E+07	1.08E+07	9.97E+07	5.12E+04	7.10E+07	2.73E+07	9.79E+07	4.40E+07	2.91E+08	1.34E+07
	China	1.08E+07	2.24E+05	7.69E+06	1.23E+05	5.28E+06	5.90E+07	6.78E+07	6.09E+07	3.23E+07	1.83E+07
	Japan	4.38E+04	7.52E+04	4.83E+04	7.58E+04	5.07E+04	3.36E+05	9.48E+04	1.81E+05	1.21E+05	1.66E+05
AOA	Africa	5.00E+08	3.73E+07	5.49E+08	7.73E+05	6.22E+08					
	China	1.34E+06	9.87E+03	1.31E+06	9.38E+03	4.18E+05					
	Japan	6.19E+05	3.42E+05	4.55E+05	4.60E+05	3.91E+05					
AOB	Africa	7.30E+05	1.15E+04	1.11E+06	1.06E+04	7.86E+05					
	China	7.34E+03	2.35E+03	2.92E+03	6.57E+02	3.70E+03					
	Japan	9.86E+03	1.88E+03	2.55E+03	1.49E+03	2.18E+03					

Supplementary Reference

Francis CA, Roberts KJ, Beman JM, Santoro AE, Oakley BB (2005) Ubiquity and diversity of ammonia-oxidizing archaea in water columns and sediments of the ocean. P Natl Acad Sci USA 102: 14683-14688. doi: 10.1073/pnas.0506625102.

Kemnitz D, Kolb S, Conrad R (2005) Phenotypic characterization of Rice Cluster III archaea without prior isolation by applying quantitative polymerase chain reaction to an enrichment culture. Environmental Microbiology 7: 553-565. doi: 10.1111/j.1462-2920.2005.00723.x.

Rotthauwe JH, Witzel KP, Liesack W (1997) The ammonia monooxygenase structural gene amoA as a functional marker: Molecular fine-scale analysis of natural ammonia-oxidizing populations. Applied and Environmental Microbiology 63: 4704-4712.

Stubner S (2002) Enumeration of 16S rDNA of Desulfotomaculum lineage 1 in rice field soil by real-time PCR with SybrGreen (TM) detection. Journal of Microbiological Methods 50: 155-164.
